# From pairwise to multiple spliced alignment

**DOI:** 10.1093/bioadv/vbab044

**Published:** 2022-01-05

**Authors:** Safa Jammali, Abigaïl Djossou, Wend-Yam D D Ouédraogo, Yannis Nevers, Ibrahim Chegrane, Aïda Ouangraoua

**Affiliations:** 1 Département D’informatique, Faculté des Sciences, Université de Sherbrooke, 2500, boul. de l'Université, Sherbrooke (Québec) J1K 2R1, Canada; 2 Département de Biochimie et de Génomique Fonctionnelle, Faculté de Médecine et des Sciences de la santé, Université de Sherbrooke, 3001, 12e avenue Nord, Sherbrooke (Québec) J1H 5N4, Canada; 3 Swiss Institute of Bioinformatics, Lausanne 1015, Switzerland; 4 Department of Computational Biology, University of Lausanne, Lausanne 1015, Switzerland; 5 Center for Integrative Genomics, University of Lausanne, Lausanne 1015, Switzerland

## Abstract

**Motivation:**

Alternative splicing is a ubiquitous process in eukaryotes that allows distinct transcripts to be produced from the same gene. Yet, the study of transcript evolution within a gene family is still in its infancy. One prerequisite for this study is the availability of methods to compare sets of transcripts while accounting for their splicing structure. In this context, we generalize the concept of pairwise spliced alignments (PSpAs) to multiple spliced alignments (MSpAs). MSpAs have several important purposes in addition to empowering the study of the evolution of transcripts. For instance, it is a key to improving the prediction of gene models, which is important to solve the growing problem of genome annotation. Despite its essentialness, a formal definition of the concept and methods to compute MSpAs are still lacking.

**Results:**

We introduce the MSpA problem and the SplicedFamAlignMulti (SFAM) method, to compute the MSpA of a gene family. Like most multiple sequence alignment (MSA) methods that are generally greedy heuristic methods assembling pairwise alignments, SFAM combines all PSpAs of coding DNA sequences and gene sequences of a gene family into an MSpA. It produces a single structure that represents the superstructure and models of the gene family. Using real vertebrate and simulated gene family data, we illustrate the utility of SFAM for computing accurate gene family superstructures, MSAs, inferring splicing orthologous groups and improving gene-model annotations.

**Availability and implementation:**

The supporting data and implementation of SFAM are freely available at https://github.com/UdeS-CoBIUS/SpliceFamAlignMulti.

**Supplementary information:**

Supplementary data are available at *Bioinformatics Advances* online.

## 1 Introduction

Alternative splicing is one of the most important mechanisms revealed in the postgenomic era (Harrow *et al.*, 2012). Over the past decade, the number of alternatively spliced genes and alternative transcripts annotated in eukaryotic organisms has increased dramatically (Zerbino *et al.*, 2018). It has now been established that alternative splicing was likely a feature of the eukaryotes’ common ancestor. Nevertheless, several questions about the evolution and conservation of sets of alternative transcripts annotated in different members of a gene family remain open (Keren *et al.*, 2010). For instance, how do new isoforms arise during evolution? Understanding the evolution of sets of alternative transcripts requires automated methods to compare sets of transcripts from homologous genes. In the past, alternative transcripts between homologous genes were compared based on pairwise spliced alignments (PSpAs). A PSpA consists in aligning a spliced RNA sequence or its DNA equivalent, the coding DNA sequence (CDS) and an unspliced DNA sequence in order to highlight homologous or equivalent exons between the aligned sequences. This is an important step for genome annotation and gene prediction using RNA-seq data or homologous gene information (Dunne and Kelly, 2018; Stanke *et al.*, 2006). For studying evolution, PSpA is also an effective method for the identification of splicing orthologous transcripts between genes, understood here as alternative transcripts of homologous genes composed of homologous exons in the same order (Jammali *et al.*, 2019; Zambelli *et al.*, 2010). Several methods have been developed to address different versions of the PSpA problem which consists in finding an optimal PSpA of two sequences given an optimization function (see Jammali *et al.*, 2019 for a review). PSpA, however, only allows two sequences to be compared at a time; therefore, it is not suited for studying alternative splicing evolution in a phylogenetic framework.

A natural generalization of PSpA to study the evolution of sets of alternative spliced RNA is multiple spliced alignment (MSpA), which is a spliced alignment of a set of spliced RNA sequences with a set of unspliced genomic sequences. Contrary to classic multiple sequence alignment (MSA), MSpA accounts for the splicing structure and the exonic structure of the input gene sequences. Like MSA that has been instrumental in better understanding the mechanisms of sequence evolution, MSpA is expected to shed light on important questions about the evolution of alternative splicing and of sets of alternative spliced RNA. For instance, the question about the conservation of the function of conserved transcripts between homologous genes remains open. MSpA will allow us to directly compare the exon architecture of alternative splicing in multiple genes and species at once, in order to answer questions about the evolution of transcripts as in (Christinat and Moret, 2013, Kuitche *et al.*, 2017). For instance, is an alternative transcript preserved between multiple genes and species? Where was it gained or lost in the evolution? How did the set of exons evolve? Moreover, like for MSA compared to pairwise sequence alignment, an MSpA of all genes and spliced RNA sequences of a gene family is expected to be more accurate than the independent PSpAs of genes and RNA sequences of the family. Furthermore, since MSpA accounts for the exon structure of genes, it will likely help improve the estimation of MSAs, as in Nord *et al.* (2018), which is critical because exon structure-related errors are pervasive in MSA.

An MSpA has important purposes other than empowering the study of the evolution of sets of alternative transcripts. Several *de novo* and homology-based gene prediction algorithms have used the idea of comparing multiple gene structures to improve gene annotation by predicting gene models for multiple homologous genes simultaneously (Dunne and Kelly, 2018; Stanke *et al.*, 2006). The MSpA framework will help to annotate new genomes by identifying exons that are homologous to exons already identified in well-annotated species, and to predict putative conserved isoforms for the newly annotated genes. Despite its essential nature, the MSpA problem has never been introduced and, to the best of our knowledge, no automated method currently exists for computing MSpAs.

In this paper, we introduce the MSpA problem as an extension of the PSpA problem that aims at finding an optimal MSpA for a set of spliced RNA sequences and a set of genes. The MSpA problem also extends a homonym but distinct problem studied in Brendel *et al.* (2004), which consists in finding an optimal alignment for a set of spliced RNA sequences and a single gene. Section 2 provides a formal definition of the MSpA problem. It also describes SplicedFamAlignMulti (SFAM), a collection of greedy heuristic methods designed to combine all PSpAs of known CDSs and gene sequences of a gene family into an MSpA. An MSpA of a gene family provided by SFAM produces a single superstructure representing the exon structure of the gene family. It separately aligns all homologous exons of all transcripts from the gene family, thus highlighting classes of conserved exons of the gene family. MSpA opens the door to broad applications for identifying and classifying splicing homology relationships between transcripts of homologous genes as in Kuitche *et al.* (2017) and to provide better gene models for genome annotation. Section 3 illustrates the utility of SFAM by comparing it to other methods on simulated gene family data to compute CDS MSAs and groups of orthologous CDSs. As no other methods exist to compute MSpA to our knowledge, the evaluation is based on MSAs and CDS orthology inferences deduced from MSpA, and the comparisons are done against state-of-the-art MSA and orthology inference methods. Lastly, we show how MSpA can be used to predict gene models by homology on a real vertebrate gene family dataset in order to improve existing genome annotations.

## 2 Materials and methods

This section introduces the MSpA problem. We then present algorithmic solutions to build an MSpA given a set of PSpAs. Lastly, we describe the experimental setup used to evaluate the methods.

### 2.1 Definition of the MSpA problem

In this section, we present formal definitions and notations of a gene model, a gene structure and spliced alignment problems.

#### 2.1.1 Gene model and gene structure

The definitions of the gene models and structure are derived from the set of RNA transcripts produced from this gene. In this study, we focused on eukaryote coding genes; the definition of gene models and structures was limited to the information about CDSs corresponding to RNA transcripts.

**Fig. 1.**
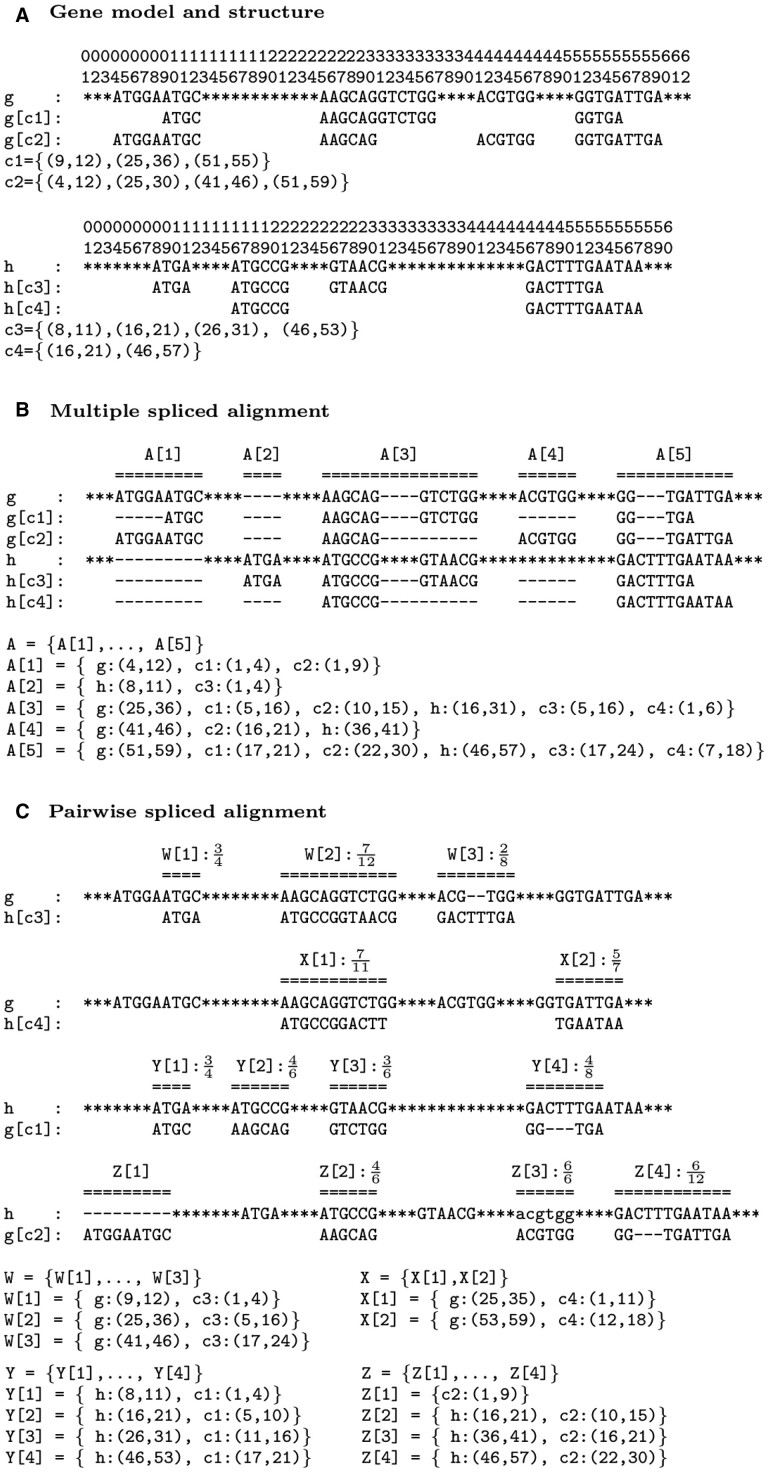
(**A**) Examples of two genes *g* and *h* having lengths 62 and 60, respectively, with sets of CDS C(g)={c1,c2} and C(h)={c3,c4}. Noncoding nucleotides in the gene sequence (i.e. introns, untranscribed and untranslated regions) are represented with the character ‘*’. For example, the sets of exons of *g* are E(g)={(4,12),(9,12),(25,30),(25,36),(41,46),(51,55),(51,59)} and the gene structure of *g* is S(g)={(4,12),(25,36),(41,46),(51,59)}. (**B**) An example of MSpA of G={g,h} and C={c1,c2,c3,c4} that is composed of 5 multiblocks. (**C**) Examples of PSpAs *W* (3 blocks), *X* (2 blocks), *Y* (4 blocks), *Z* (4 blocks) for the pairs of CDS and gene $\left(c_{3},g),\;(c_{4},g),\;(c_{1},h\right)$ and $\left(c_{2},h\right)$, respectively. All blocks are conserved blocks, except Z[1], which is a deleted block. For each conserved block, the PID of the segment alignment is depicted

A *gene sequence* is a DNA sequence on the alphabet of nucleotides Σ={A,C,G,T}. Given a gene sequence *g* of length *n*, an *exon* of *g* is a segment represented by the pair of its start and end locations on *g*, (*a*, *b*) such that 1≤a≤b≤n. A *gene model* of *g* is a representation of a CDS *c* of *g* as a chain of exons of *g*, $c=\{(a_{1},b_{1}),\ldots ,(a_{j},b_{j})\}$, such that for any two successive exons (*a_i_*, *b_i_*) and $\left(a_{i+1},b_{i+1}\right)$ in *c*, we have $b_{i}<a_{i+1}$. The *i*th exon of a CDS *c* is denoted by c[i]. We denote by C(g) the set of alternative CDS of a gene *g*. The set of all exons of *g* composing its CDS is denoted by $E(g)=\underset{c\in C(g)}{\cup }c$. The *gene structure* of *g*, denoted by S(g) is the chain of nonoverlapping segments of *g* obtained by merging all overlapping exons of E(g), and ordering the resulting segments according to their locations in *g* (see Fig. 1A).

The sequence of a CDS *c* of *g*, denoted by g[c], is the concatenation of the sequences of gene exons composing *c*. An exon of a CDS g[c] of length *m* is a segment represented by the pair of its start and end locations on g[c], (*k*, *l*) such that 1≤k≤l≤m. We denote by E(g[c]) the set of all exons composing a CDS g[c]. For instance, the example depicted in Figure 1A shows that g[*c*_1_] = ATGCAAGCAGGTCTGGGGTGA has a length of 21 with a set of exons E(g[c1])={(1,4),(5,16),(17,21)}. Given a CDS g[c] of length *m* and a location *k* such that $1\leq k\leq m,\;\mathrm{gpo}s_{c\to g}(k)$ denotes the location on gene *g* corresponding to the location *k* on g[c]. For instance in Figure 1A, $\mathrm{gpo}s_{c1\to g}(1)=9,\;\mathrm{gpo}s_{c1\to g}(5)=25$.

#### 2.1.2 Spliced alignment problems

A PSpA is an alignment of a CDS and a gene sequence that accounts for the splicing structure of sequences and allows identification of homologous exon sequences. In Jammali *et al.* (2019), a PSpA was formulated as a chain of blocks corresponding to pairwise sequence alignments of segments of the CDS with segments of the gene. This formulation makes it possible to define various versions of the PSpA problem under a unified framework. It also allows to highlight the macroscopic alignment at the level of the splicing (exon–intron) structure, rather than the microscopic alignment at the level of nucleotide, since we focus here on the splicing trends and exon usage differences between different gene family members. Herein, the concept of PSpA and the formulation from Jammali *et al.* (2019) are extended to define MSpAs. An MSpA is an alignment of a set of CDSs with a set of gene sequences that accounts for the splicing structure of sequences and makes it possible to highlight homologous exons.

Definition 1 [multiple spliced alignment (MSpA)] An MSpA of a set of CDSs C and a set of genes G from the same gene family is represented as a chain A={A[1],…,A[n]} of maps called multiblocks such that A[i] denotes the *i*th multiblock of *A*. Each multiblock A[i] is a map whose key set, denoted by key(A[i]), is a subset of C∪G, and A[i] associates each CDS or gene x∈key(A[i]) to a segment on the sequence of *x* represented by the pair $\left(s_{i}^{x},e_{i}^{x}\right)$ of its start and end locations. An MSpA A={A[1],…,A[n]} of a set of CDSs C and a set of genes G must satisfy the following four conditions (see Fig. 1B for an illustration):

1. For any multiblock A[i], we must have | key(A[i]) |>0.
2. For any CDS or gene x∈C∪G and any two multiblocks $A[i_{1}]$ and $A[i_{2}]$ such that $i_{1}<i_{2}$ and $x\in \mathrm{key}(A[i_{1}])\cap \mathrm{key}(A[i_{2}])$, we must have $e_{i_{1}}^{x}<s_{i_{2}}^{x}$, i.e. the end location of the segment of *x* in $A[i_{1}]$ is before the start location of the segment of *x* in $A[i_{2}]$.
3. For any CDS c∈C, the set of segments of *c* induced by *A*, $\left\{(s_{i}^{c},e_{i}^{c})\;|\;1\leq i\leq n\;\mathrm{and}\;c\in \mathrm{key}(A[i])\right\}$ covers entirely *c*.
4. For any multiblock A[i] and any CDS c∈C of a gene g∈G such that c∈key(A[i]), we must have g∈key(A[i]) and the interval $\left(\mathrm{gpo}s_{c\to g}(s_{i}^{c}),\mathrm{gpo}s_{c\to g}(e_{i}^{c})\right)$ is included in the interval $\left(s_{i}^{g},e_{i}^{g}\right)$, i.e. the segments of *c* and *g* in A[i] must be consistent.

Each multiblock $A[i]=\{x:(s_{i}^{x},e_{i}^{x})\;|\;x\in \mathrm{key}(A[i])\}$ represents an alignment of the set of segments associated with elements of key(A[i]). It represents a set of conserved exon segments. The size of a multiblock A[i] is the size of its key set key(A[i]).

Given two multiblocks $a=\{x:(s_{a}^{x},e_{a}^{x})\;|\;x\in \mathrm{key}(a)\}$ and $b=\{x:(s_{b}^{x},e_{b}^{x})\;|\;x\in \mathrm{key}(b)\}$, we say that *a* is *consistent* with *b*, if one of the following conditions is satisfied:

- for any $x\in \mathrm{key}(a)\cap \mathrm{key}(b),\;e_{a}^{x}<s_{b}^{x}$, i.e. *a* is located before *b* or
- for any $x\in \mathrm{key}(a)\cap \mathrm{key}(b),\;e_{b}^{x}<s_{a}^{x}$, i.e. *a* is located after *b* or
- for any x∈key(a)∩key(b), the segments $\left(s_{a}^{x},e_{a}^{x}\right)$ and $\left(s_{b}^{x},e_{b}^{x}\right)$ overlap, i.e. *a* overlaps *b*.

Given an MSpA *A* of C∪G and a multiblock *a* on C∪G, the multiblock *a* is consistent with *A* if for any multiblock A[i] of *A*, *a* is consistent with A[i].

Definition 2 [pairwise spliced alignment (PSpA)] A PSpA is an MSpA of a single CDS and a single gene. In this case, the multiblocks of the alignment are called blocks (see Fig. 1C for an illustration).

Let *X* be a PSpA of a CDS c∈C and a gene g∈G. A block X[i] of *X* is a *conserved block* if *c* and *g* belong to key(X[i]) Otherwise, if c∈key(X[i]) and g∈key(X[i]), then X[i] is called a *deleted block* (see Fig. 1C for an illustration).

An MSpA A={A[1],…,A[n]} of a set of CDSs C and a set of genes G induces a PSpA $A_{c,g}$ of each pair of CDS c∈C and gene g∈G. The induced PSpA is obtained by first reducing *A* to the chain of multiblocks A[i] such that c∈key(A[i]), and then removing all entries except *c* and *g* from the multiblocks. For instance, for the MSpA depicted in Figure 1B, the induced PSpA of CDS c3 and gene *g* is composed of 3 blocks, $A_{c3,g}=\{A_{c3,g}[1],A_{c3,g}[2],A_{c3,g}[3]\}$ that are reductions of A[2], A[3] and A[5].

Various definitions of similarity scores for PSpAs have been considered to define various versions of the PSpA problem (Jammali *et al.*, 2019). Let S be a scoring function that associates any PSpA *P* of a CDS sequence *c* and a gene sequence *g* with a similarity score S(P). The general MSpA problem is defined as follows:

#### 2.1.3 Multiple spliced alignment problem (MSAP_I)

**Input**: A set of CDSs C; a set of genes G.

**Output**: An MSpA *A* of C on G that maximizes the sum of the scores of induced PSpAs: $\sum_{(c,g)\in C\times G}S(A_{c,g})$.

There exist several methods for computing PSpAs between a gene and a CDS (Jammali *et al.*, 2019). Considering this, if a set of optimal PSpAs of all pairs of a CDS and gene in C×G is available, the MSAP can be reduced to one of optimally combining the PSpAs into an MSpA, as follows:

#### 2.1.4 Multiple spliced alignment problem (MSAP_II)

**Input**: A set of CDSs C; a set of genes G; a set of PSpAs $X=\{X_{c,g}\;|\;(c,g)\in C\times G\}$ for all pairs in C×G.

**Output**: An MSpA *A* of C and G that maximizes the number of blocks of pairwise alignments in X that are included in multiblocks of *A*.

### 2.2 The SFAM algorithms

We now describe our heuristic algorithms for building an MSpA of a set of CDSs C and a set of genes G, given a set of PSpAs $X=\{X_{c,g}\;|\;(c,g)\in C\times G\}$ for all pairs in C×G (MSAP_II problem). MSA methods usually make use of greedy heuristics, including the most widely used progressive alignment strategy (Feng and Doolittle, 1987). The progressive alignment strategy consists in first building a guide tree using pairwise comparison information. The sequences and alignments are then merged progressively from the leaves to the root of the guide tree. Progressive alignment has the classic local-optimum limitations of greedy algorithms. One way to address this problem is to use consistency scoring that was pioneered by T-Coffee (Notredame *et al.*, 2000). The consistency-based strategy consists in evaluating the degree to which the alignment of two residues or segments in a precomputed pairwise alignment is supported by other precomputed pairwise alignments. Using the consistency-based strategy in a progressive alignment approach can account for the information of all pairwise alignments at each stage of the alignment in order to avoid the classic limitations of progressive alignment.

The first algorithm named SplicedFamAlignMulti_tcoffee (SFAM_tcoffee) uses a consistency-based progressive alignment that relies on the use of the T-Coffee alignment package (Notredame *et al.*, 2000).

The second algorithm named SplicedFamAlignMulti_mblock (SFAM_mblock) also uses a greedy approach that consists in first precomputing a set of candidate multiblocks by assembling aligned pairwise blocks that share identical segment. The candidate multiblocks are then sorted according to their size, including multiblocks one-by-one into a growing consistent set of multiblocks. We call this approach the greedy multiblock approach. The rationale behind this approach is that a candidate multiblock of large size is supported by a high number of pair blocks, and is less likely to be a random artifact than a multiblock containing a small number of segments. Like the consistency-based progressive alignment, the greedy multiblock approach also takes into account the information of all pairwise alignments at each stage of the alignment by considering candidate multiblocks instead of pair blocks.

### 2.3 Spliced alignment graph

**Fig. 2.**
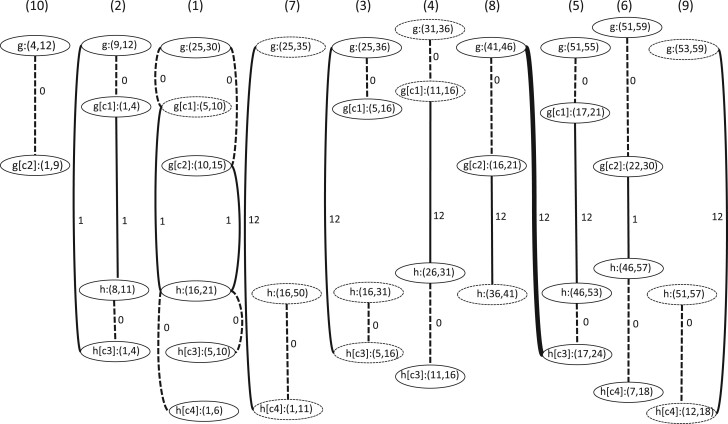
Spliced alignment graph graph(X) of the PSpA depicted in Figure 1C. Block edges are represented as solid lines, and CDS edges as dashed lines. The value of *connect*(*e*) is indicated for each edge *e*. Segments that do not correspond to exons in CDS or gene sequences are represented as dashed lines. The edge represented by a thicker line is removed at Step 3 of the graph-based MSpA algorithm to obtain graph′(X). The connected components of graph′(X) are numbered in decreasing order of size

The two algorithms SFAM_tcoffee and SFAM_mblock make use of a spliced alignment graph, denoted by graph(X), that represents a set of PSpAs $X=\{X_{c,g}\;|\;(c,g)\in C\times G\}$ (see Fig. 2 for an illustration).

Definition 3 (spliced alignment graph) Let $X=\{X_{c,g}\;|\;(c,g)\in C\times G\}$ be a set of PSpAs. For any CDS c∈C of a gene g∈G, and any gene h∈G, for any conserved block $X_{c,h}[i]=\{h:(s_{i}^{h},e_{i}^{h}),c:(s_{i}^{c},e_{i}^{c})\}$ of $X_{c,h}$, the spliced alignment graph graph(X) represents the segments $\left(s_{i}^{h},e_{i}^{h}),\;(s_{i}^{c},e_{i}^{c}\right)$ and $\left(\mathrm{gpo}s_{c\to g}(s_{i}^{c}),\mathrm{gpo}s_{c\to g}(e_{i}^{c})\right)$ as vertices with an edge between segments $\left(s_{i}^{h},e_{i}^{h}\right)$ and $\left(s_{i}^{c},e_{i}^{c}\right)$ that represents the conserved block, and an edge between segments $\left(s_{i}^{c},e_{i}^{c}\right)$ and $\left(\mathrm{gpo}s_{c\to g}(s_{i}^{c}),\mathrm{gpo}s_{c\to g}(e_{i}^{c})\right)$ that represents the alignment of CDS segment $\left(s_{i}^{c},e_{i}^{c}\right)$ on the corresponding gene segment. The edges of graph(X) that represent conserved blocks are called block edges, while edges that represent alignments of CDS segments of their corresponding genes are called CDS edges. Note that if *g* = *h*, then $\left(s_{i}^{h},e_{i}^{h}\right)$ = $\left(\mathrm{gpo}s_{c\to g}(s_{i}^{c}),\mathrm{gpo}s_{c\to g}(e_{i}^{c})\right)$, and the conserved block $X[i]=\{h:(s_{i}^{h},e_{i}^{h}),c:(s_{i}^{c},e_{i}^{c})\}$ induces a single edge in graph(X) that is a CDS edge.

A fully detailed description of each step of the algorithms is provided in the Supplementary Material. Below, we briefly describe the steps in each algorithms. See Supplementary Figure S1 for an overview of the two methods.

### 2.4 T-Coffee-based MSpA

The SFAM_tcoffee algorithm follows a consistency-based progressive alignment strategy that relies on the use of the T-Coffee alignment package (Notredame *et al.*, 2000). The T-Coffee algorithm implements the consistency-based strategy by generating and making use of a library of pairwise residue matches obtained from pairwise alignments. Each pair of aligned residues in the primary library is first weighted using the sequence identity of the pairwise alignment from which it comes. This is done in order to reflect the correctness of their alignment in a pairwise context. Next, the library is extended in order to include pairs of residues between two sequences induced by a third sequence. The weights of pairs of residues are also extended such that the final weight of a pair of residues reflects the similarity of their sequences as well as the consistency of the residue pair with all other residue pairs in the library. The SFAM_tcoffee algorithm is composed of three steps:

1. **Generate the primary library of residue pairs**: SFAM_tcoffee provides two ways to generate the primary library of residue pairs.
• The first method named SFAM_tcoffee_p (p for pairwise) makes direct use of the blocks of the PSpAs given as input to SFAM_tcoffee to extract the pairs of aligned residues between gene sequences.
• The second method named SFAM_tcoffee_m (m for multiple) makes use of the alignment graph graph(X). For each connected component *cc* of graph(X), an MSA is computed for the set of gene segments contained in *cc*, and pairs of aligned residues between gene sequences are extracted from the MSA.
2. **Compute anMSA*M* of**G: An MSA of gene sequences is computed using the T-Coffee algorithm and the library of residue pairs computed at Step 1.
3. **Compute anMSpAof**C∪G**given theMSA*M* of**G: The MSA computed at Step 2 is used to partition the set of all segments of all gene structures into groups of aligned segments that correspond to multiblocks of an MSpA.

### 2.5 Multiblock greedy MSpA

The SFAM_mblock is based on the alignment graph graph(X). It aims at defining the multiblocks of the MSpA as the connected components of a graph obtained after modification of graph(X). The algorithm compromises four steps:

1. **Compute the alignment graph** graph(X) .
2. **Weight the edges of** graph(X) : Two scores denoted by *PID*(*e*) and *connect*(*e*) are assigned to each edge *e* of graph(X). *PID*(*e*) is the percent sequence identity (PID) of the pairwise sequence alignment represented by *e*. *connect*(*e*) evaluates the strength of the connectivity between the two vertices connected by *e*. The two scores serve as confidence scores for the segment alignment represented by *e*.
3. **Split connected components of** graph(X) : For each connected component *cc* of graph(X), if *cc* contains two vertices corresponding to two nonoverlapping segments of a sequence *x*, then *cc* cannot correspond to a multiblock, since a multiblock contains at most one segment from each sequence x∈C∪G. In this case, based on the confidence scores computed in the preceding step, a set of low confidence edges are removed from *cc* to disconnect the two vertices.
4. **Build the** **MSpA** **in a progressive manner**: The connected components of graph′(X) are considered as candidate multiblocks. For each connected component *cc* of graph′(X), a candidate multiblock composed of the segments (vertices) in *cc* is built. The resulting set M of candidate multiblocks is ordered by decreasing multiblock size. The MSpA *A* is initialized to an empty chain, and candidate multiblocks that are consistent with *A* are progressively added to A.

### 2.6 Computation of multiple CDS alignments, CDS orthology groups and putative CDS given an MSpA

**Fig. 3.**
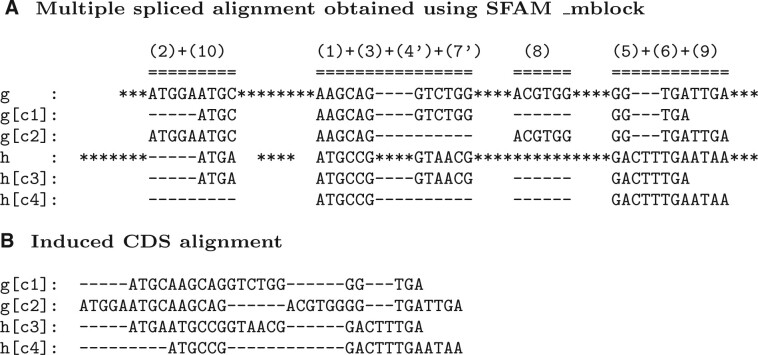
(**A**) The MSpA of the set of genes G={g,h} and the set of CDSs C={c1,c2,c3,c4} from Figure 1A, resulting from the application of Algorithm SFAM_mblock. (**B**) The MSA of C={c1,c2,c3,c4} induced by the MSpA

Let A={A[1],…,A[n]} be an MSpA of C∪G. A multiple CDS alignment, CDS orthology groups and putative CDS can be deduced from *A* as follows:

**Computing an** **MSA** **of** C : An MSA is first computed for each multiblock A[i] while forcing the correct alignment of any CDS segment on its corresponding gene segment. The resulting alignments are then reduced to C, and concatenated to obtain an MSA of C (see Fig. 3 for an illustration).

**Computing CDS orthology groups**: Given two CDSs *c*_1_ and *c*_2_ of C from two distinct genes of G, we define *c*_1_ and *c*_2_ as orthologous CDS if:

- for any multiblock $A[i]\in A,\;c_{1}\in \mathrm{key}(A[i])$ if and only if $c_{2}\in \mathrm{key}(A[i])$, and;
- if $c_{1}\in \mathrm{key}(A[i])$ then $(e_{i}^{c_{1}}-s_{i}^{c_{1}})-(e_{i}^{c_{2}}-s_{i}^{c_{2}})\;\%\;3=0$.

In other terms, *c*_1_ and *c*_2_ are orthologs if they have segments in the same multiblocks, and the lengths of each pair of segments of *c*_1_ and *c*_2_ that belong to the same multiblock are congruent modulo 3. These conditions ensure that *c*_1_ and *c*_2_ have the same splicing structure, and are translated in the same reading frame in order to yield similar protein sequences. For instance, in the MSpA depicted in Figure 3, *g*[*c*_1_] and *h*[*c*_3_] are orthologs. Given the orthology relation defined on the set of CDSs C, we extend it to a reflexive, transitive and symmetric relation (i.e. an equivalence relation), and the resulting equivalence classes are defined as CDS orthology groups.

**Predicting gene models by homology**: Let *c*_1_ be a CDS of a gene g∈G, and h∈G be a gene distinct from *g*. A putative CDS *c*_2_ can be predicted for *h* if:

- for any multiblock A[i]∈A, c1∈key(A[i]) if and only if h∈key(A[i]), and;
- if $c_{1}\in \mathrm{key}(A[i])$ then $(e_{i}^{c_{1}}-s_{i}^{c_{1}})-(e_{i}^{h}-s_{i}^{h})\;\%\;3=0$.

The putative CDS *c*_2_ is obtained by concatenating the set of segments of *h* contained in multiblocks that contains segments of *c*_1_, $\left\{(s_{i}^{h},e_{i}^{h})\;|\;1\leq i\leq n\;\mathrm{and}\;c_{1}\in \mathrm{key}(A[i])\right\}$. The resulting sequence *c*_2_ is a predicted CDS for *h*, if it is not already a CDS of *h*, and it does not contain any STOP codons. It is easy to see that *c*_1_ and the predicted CDS *c*_2_ will then be orthologs according to the definition provided above. For instance, in the MSpA depicted in Figure 3, a CDS g[c3] = AAGCAGGTCTGG|GGTGATTGA orthologous to h[c4] and composed of two exons can be predicted.

### 2.7 Experimental setup

#### 2.7.1 Dataset

To evaluate the performance and show the utility of SFAM, we used three datasets of 20 simulated gene families, and a dataset of 20 real gene families from the Ensembl database ([Bibr vbab044-B26][Bibr vbab044-B26]).

##### 2.7.1.1 Simulated data

Since true MSpAs of real gene families were not available for validating our MSpA method, we generated three datasets of 20 simulated gene families with gene sequences and CDSs using SimSpliceEvol (Kuitche *et al.*, 2019). SimSpliceEvol relies on a model of gene splicing structure evolution and the evolution of transcript sets through alternative splicing events. It takes as input a gene tree with branch lengths representing evolutionary rates. It relies on empirically determined distributions of the number of exons per transcript, the number of alternative transcripts per gene, the length of exon segments, the length of intron segments in 189 eukaryotic species to generate realistic gene sequences. It generates an ancestral gene sequence at the root of the tree with exon–intron structure and its set of CDSs. It makes this gene evolve along the branches of the gene tree to generate a gene sequence and a set of CDSs for each node of the tree. The root gene sequence, its CDS, and its exon–intron structure are generated based on parameters learned from a dataset of 10 000 vertebrates genes from amniote species from the Ensembl database (Zerbino *et al.*, 2018). The evolution simulated along branches of the tree accounts for two levels of evolution. First, at the level of genes, the evolution model includes exon duplication; gain and loss events that can modify the exon–intron structure of genes and amino-acid (resp. nucleotide) insertion, deletion and substitution events that can modify the sequence of coding exons (resp. noncoding introns). For sequence evolution and indel model, SimSpliceEvol uses the simulation models in Strope *et al.* (2009). In indel-Seq-Gen 2, the branch lengths given in the guide tree determine the rates of evolution, which are introduced based on models of continuous substitution evolution processes with lineage- and site-specific conservation as well as heterogeneous evolution. For realistic sequence evolution, it also uses an indel model that relies on a noncontinuous process with dynamic sequence length adjustment, event tracking and empirical length distribution, with respect to different sequence and functional constraints placed on insertion and deletion processes. Second, at the level of transcripts, the evolution model includes isoform creation and loss events, as well as alternative splicing events that can modify the sets of transcripts (CDSs) generated by genes. Using SimSpliceEvol, we generated three sets of 20 gene families: a first set called *Small* using as input gene tree a species tree of 5 primates with low evolutionary rates on the tree branches, a second set called *Medium* with medium evolutionary rates using a tree of 5 primates and rodents, and a third set called *Large* with high evolutionary rates using a tree of 5 amniote species. The input trees used for the simulations were retrieved from the Ensembl Compara database (Zerbino *et al.*, 2018). They are described in the Supplementary Material. The simulation using the guide trees does not follow a strict molecular clock, but rather relies on a model of continuous and heterogeneous substitution evolution for realistic sequence evolution (see description of indel-Seq-Gen 2). Each of the three sets contains 20 simulated gene families with 5 genes and 5–11 CDSs in total. For each family, SimSpliceEvol provides the true MSA of all CDS and gene sequences generated. SimSpliceEvol also outputs all groups of splicing orthologs that are groups of CDSs descending from the same ancestral CDS without any alternative splicing events in their evolutionary history from the ancestral CDS. In other words, two CDSs from two different genes are orthologs and belong to the same group if their sets of exons descend from the same ancestral set of exons and only sequence mutations events have occurred in the evolution of exons from their common ancestor. Table 1 shows the detailed description of the simulated datasets.

**Table 1.** Description of the three simulated datasets: for the average measures, the standard deviations are also given in parentheses (PID)

	Small	Medium	Large
Number of families	20	20	20
Number of genes per family	5	5	5
Avg. number of CDS	8.8	8.55	9.35
	(3.02)	(3.04)	(2.70)
Avg. CDS length	802.65	820.49	778.13
	(204.41)	(288.37)	(281.77)
Avg. gene length	2358.71	2724.82	2418.22
	(713.76)	(813.09)	(601.44)
Avg. pairwise PID (%)	71.24	54.39	41.61
	(4.36)	(2.52)	(2.4)

##### 2.7.1.2 Real dataset

We randomly selected 20 gene families from the Ensembl database containing genes from 6 amniote species: *human*, *mouse*, *dog*, *dingo*, *cow* and *chicken*. For each gene family, we removed all genes except those from the 6 selected species. For each gene family, the dataset contains the sequences of the remaining genes from 6 species, and all CDSs from these genes. For each gene family, the sets of CDSs from genes were retrieved for releases 97 and 98 of the Ensembl database, because, in release 98 a new gene annotation of *dog* was provided, leading to an increase from 19 857 to 20 257 coding genes, and from 39 074 to 60 994 gene transcripts. This dataset was used to evaluate the ability of SpliceFamAlignMulti to predict the new dog CDSs present in release 98, based on the spliced alignments computed on the data from release 97 of Ensembl. Table 2 shows the detailed description of the real dataset.

**Table 2.** Description of the real dataset of 20 gene families (PID)

Gene family (tree) ID	No. of total genes	No. of total CDS (97)–(98)	No. of dog genes	No. of dog CDS (97)–(98)	PID
ENSGT00530000063205	19	42–45	3	3–6	0.098
ENSGT00390000008371	12	27–28	2	2–3	0.604
ENSGT00390000000715	6	12–15	1	1–4	0.474
ENSGT00940000157909	6	31–35	1	1–5	0.623
ENSGT00530000063187	17	28–30	3	3–5	0.177
ENSGT00950000182978	24	88–103	4	4–19	0.106
ENSGT00950000182681	43	79–89	7	9–19	0.147
ENSGT00950000182875	37	57–63	5	4–10	0.112
ENSGT00950000182728	35	75–83	6	6–14	0.073
ENSGT00950000182931	24	73–83	4	5–15	0.168
ENSGT00950000182705	39	111–115	6	5–9	0.087
ENSGT00530000063023	23	41–46	4	6–11	0.103
ENSGT00940000153241	14	16–19	3	3–6	0.06
ENSGT00950000182956	23	56–71	4	5–20	0.257
ENSGT00950000182783	29	72–77	4	4–9	0.045
ENSGT00950000183192	19	69–72	3	3–6	0.051
ENSGT00950000182727	30	73–84	5	6–17	0.047
ENSGT00390000004965	6	7–8	1	1–2	0.89
ENSGT00390000003967	6	9–12	1	1–4	0.799
ENSGT00390000005532	6	12–13	1	1–2	0.545

#### 2.7.2 Evaluated methods

To the best of our knowledge, SFAM has no competing methods for MSpAs. For this reason, the performance of SFAM algorithms was compared to the performance of popular MSA methods, using the resulting multiple CDS alignments. This is pertinent because the quality of a multiple CDS alignment induced by an MSpA is directly related to the quality of the MSpA. The SFAM algorithms were also compared to sequence clustering and orthology group inference methods used to compute CDS orthology groups within a gene family.

##### 2.7.2.1 MSA methods

SFAM was compared to popular MSA methods including MUSCLE (Edgar, 2004), MAFFT (Katoh and Standley, 2013), MACSE (Ranwez *et al.*, 2018), CLUSTAL_O (Sievers *et al.*, 2011), PRANK (Löytynoja and Goldman, 2005, 2008), T-Coffee (Notredame *et al.*, 2000) and Mirage (Nord *et al.*, 2018). MUSCLE (Edgar, 2004) is a multiple sequence aligner that uses the log-expectation score to speed up its progressive alignment protocol. MAFFT (Katoh and Standley, 2013) is an MSA program based on the identification of homologous regions by the fast Fourier transform. MACSE (Ranwez *et al.*, 2018) is a multiple sequence aligner that accounts for the underlying codon structure of protein-coding nucleotide sequences. CLUSTAL_O (Sievers *et al.*, 2011) uses guide trees and an HMM profile-profile approach to compute MSAs. PRANK (Löytynoja and Goldman, 2005, 2008) is a probabilistic MSA program, based on maximum likelihood methods that take into account the evolutionary distances between sequences. T-Coffee (Notredame *et al.*, 2000) is a multiple sequence aligner that uses a consistency-based progressive strategy that relies on a library of pairwise residue matches obtained from pairwise alignments. Mirage (Nord *et al.*, 2018) is a multiple sequence aligner for alternatively spliced protein isoforms that maps protein sequences to their corresponding genomic sequences, and then aligns the isoforms based on these mappings. The three algorithms of SFAM (SFAM_tcoffee_p, SFAM_tcoffee_m and SFAM_mblock) were included in the comparison. The default parameters were used for all the methods included in the comparison.

##### 2.7.2.2 Sequence clustering and orthology group inference methods

SFAM was compared to the following sequence clustering methods that can be used to compute CDS orthology groups within a gene family: Cluss (Kelil *et al.*, 2008), OrthoFinder (Emms and Kelly, 2019) and SplicedFamAlign (SFA; Jammali *et al.*, 2019). Cluss (Kelil *et al.*, 2008) is a fast alignment-free method for clustering protein families. OrthoFinder (Emms and Kelly, 2019) is a widely used alignment-based method that infers orthogroups of coding genes by solving the gene length bias in orthogroup inference. SFA (Jammali *et al.*, 2019) is the PSpA methods on which SFAM algorithms are based. SFA infers CDS orthology groups based on the set of all PSpAs within a gene family. The default parameters were used for all the methods.

#### 2.7.3 Performance metrics

The following performances metrics were used to compare the methods included in the analyses.

##### 2.7.3.1 Performance for MSAs

Based on the true MSAs of the CDS of each family of the simulated datasets, the precision, recall, *F*-score, and computing time for each method and each gene family were computed. The *precision* is the fraction of nucleotide pairs in the estimated alignment that are also in the true alignment. The *recall* is the fraction of nucleotide pairs in the true alignment that are also in the estimated alignment. The *F-score* is the harmonic mean of precision and recall. In order to account for the fact that the methods of SFAM and Mirage use the gene sequences as guides to improve CDS alignment, we considered two sets of results. The first set of results was obtained by directly aligning only the CDSs using the other MSA methods (MUSCLE, MAFFT, MACSE, CLUSTAL_O, PRANK, T-Coffee). The second set of results was obtained by aligning the gene sequences and the CDSs using the other alignment methods (MUSCLE_G, MAFFT_G, MACSE_G, CLUSTAL_O_G, PRANK_G, T-Coffee_G), and then computing the CDS alignments induced by these whole MSAs. The precision, recall, *F*-score and computing time were computed for each of these sets of results. For SFAM, the computing time for PSpAs is included.

##### 2.7.3.2 Performance for CDS orthology group inference

Based on the true splicing orthologous groups of the CDS for each family of the simulated datasets, the Rand index (RI) and the computing time for each method and each gene family were calculated. The RI is the fraction of CDS pairs that have the same relation in the estimated clustering and the true clustering, either in the same cluster or in different clusters.

##### 2.7.3.3 Performance for gene-models prediction

Based on the results of the comparison of MSA methods and the comparison of sequence clustering and orthology group inference methods, only the best performing algorithm of SFAM SFAM_mblock was used in predicting new gene models by homology. For each gene family of the real dataset, we considered the set *T* (for true) of dog CDSs present in release 98 of the Ensembl database, but absent in release 97, and the set *P* (for predicted) of dog CDSs predicted by SpliceFamAlignMulti (SFAM_mblock) based on data from release 97. We computed the precision, recall and *F*-score of the prediction. The *precision* is the fraction of CDSs in *P* that correspond to CDS *T*. The *recall* is the fraction of CDS in *T* that corresponds to CDS in *P*, and the *F-score* is again the harmonic mean of precision and recall. For each gene family, we considered two methods to define the correspondence between CDSs of *T* and *P*. The more stringent method defines two CDSs of *T* and *P* as matching if they are 100% identical. The second less stringent method defines two CDSs of *T* and *P* as matching if they are >90% identical and reciprocal best hits. For this second method, the PID is computed based on a pairwise alignment of the CDS that is computed using a global alignment method, FsePSA (Jammali *et al.*, 2017), a pairwise alignment for CDSs that simultaneously considers nucleotide and amino-acid sequences and accounts for frameshift translation initiation and length.

## 3 Results and discussion

### 3.1 Performance for MSA

**Fig. 4.**
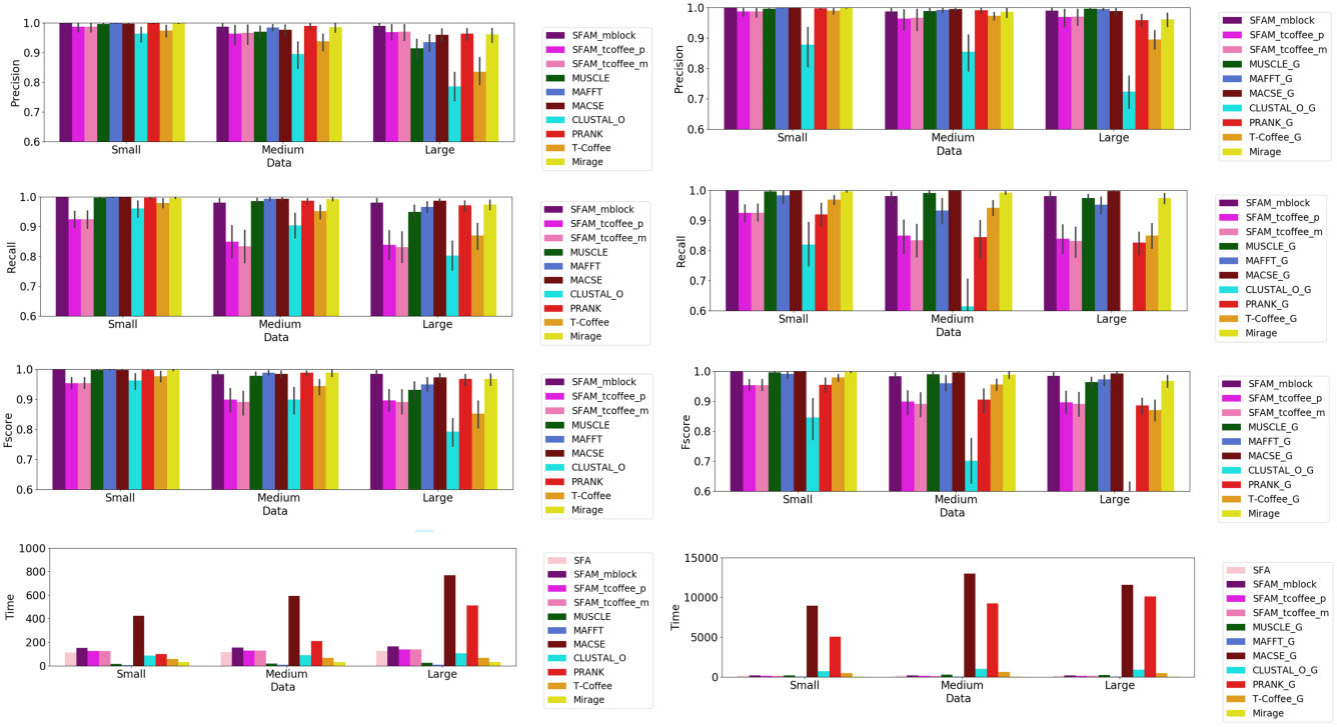
Precision, recall and *F*-score measures with their standard deviations for all compared methods for the MSAs of CDSs (left column). The first set of results obtained by aligning only the CDSs using the MSA methods (MUSCLE, MAFFT, MACSE, CLUSTAL_O, PRANK, T-Coffee) (right column). The second set of results obtained by aligning the gene sequences and the CDSs, and then extracting the induced CDSs alignment. The sum of execution times in second for all methods and all simulated datasets are also given

The results of the comparison with MSA methods are shown in Figure 4.

#### 3.1.1 Comparison of Graph and T-Coffee-based MSpA

Comparing the different versions of our SFAM method in terms of precision, recall and *F*-score, SFAM_mblock performed best for all classes of sequence similarity (Fig. 4, left). Of the two versions of SFAM based on T-Coffee, SFAM_tcoffee_p appears to perform slightly better than SFAM_tcoffee_m, but the difference is not significant. This shows the superiority of the strategy that consists in macroscopically assembling pairwise blocks into multiple blocks without relying on sequence alignment (SFAM_mblock) over the strategy that first computes the sequence alignment and then computes the multiple blocks of the MSpA based on the sequence alignment (SFAM_tcoffee). It is worth noting that the low performance of the SFAM_tcoffee methods might well be related to the low performance of T-Coffee observed and discussed in the next paragraph. As SFAM_mblock performed best, we excluded SFAM_tcoffee_p and SFAM_tcoffee_m from the rest of the analysis, and only considered SFAM_mblock for comparison with the other methods.

#### 3.1.2 Comparison of SFAM_mblock MSpA with MSA methods

In terms of precision, SFAM_mblock, MUSCLE, MAFFT, MACSE, PRANK and Mirage performed well with precision rates higher than 0.9 for all classes of sequence similarity (Fig. 4, left). Among these methods, SFAM_mblock was the most robust across the three classes of sequence similarity, and had a precision rate close to 1, the best among all the methods. In contrast, the precision rates of CLUSTAL_O and T-Coffee decreased significantly from the Small to the Large datasets. In terms of recall, we observed the same trend as for the precision rate. CLUSTAL_O and T-Coffee performed least well, while the other methods performed well with a recall rate higher than 0.9 for all classes of sequence similarity. Unlike the case of precision, however, no methods significantly outperformed the others in terms of recall across the three classes of sequence similarity. As expected, the *F*-score measures reflect what was observed with precision and recall, showing good and comparable performances for SFAM_mblock, MUSCLE, MAFFT, MACSE, PRANK and Mirage for all datasets with values higher than 0.9. SFAM_mblock was the most robust across the three classes of sequence similarity, trailed closely by MACSE, PRANK and Mirage. These results show that SFAM_mblock produced sequence alignments at least as accurate as the compared multiple sequence aligners.

#### 3.1.3 Comparison when adding the gene-sequence information for other methods

Unlike the other methods, SFAM and Mirage use the gene-sequence information. To evaluate the advantage provided by the inclusion of gene-sequence information, they were added as input for the other methods. Figure 4 right provides the comparison results. As expected, adding the gene sequences in the alignment improves the precision of MUSCLE, MAFFT, MACSE and T-Coffee. As for the recall measure, however, only MACSE showed slightly higher for the rest of the analysis when gene sequences were considered in the alignment. The recall measures for MAFFT and especially PRANK significantly decreased. Lastly, CLUSTAL_O performed clearly worse, in terms of both precision and recall, when the gene sequences were added to the alignment. Overall, when taking account of the gene sequences, MAFFT, MACSE, MUSCLE and Mirage achieve good and comparable performance. SFAM_mblock and MACSE performed best, followed by Mirage, MAFFT and Muscle. These results show that the inclusion of gene-sequence information improved the accuracy of most of the methods, especially in terms of precision but SFAM_mblock stood out as among the methods with the best performance. Moreover, including gene-sequence information made the other methods much more time-consuming, as discussed in the next paragraph.

#### 3.1.4 Comparison of execution times

All the methods ran on the same platform using the same environment (Fig. 4, left). MACSE and PRANK had much higher execution times than the other methods, with comparable execution times of <200 s on all datasets. Nevertheless, the SFAM algorithms had longer execution times than MUSCLE, MAFFT, CLUSTAL and Mirage. This is probably due to the inclusion of gene sequences in the inputs of SFAM, since when gene sequences were added for other methods, their execution times were at least as high for SFAM (Fig. 4, right). When the gene sequences were added, the execution times of MACSE and PRANK the two most computationally intensive were even greater compared to the other methods. This can be explained by the time complexities of MACSE and PRANK, which increase more rapidly as the length of the input sequences increases, because gene sequences are much longer than CDSs.

#### 3.1.5 Conclusion on the performance for MSA

The results of the comparison of the different versions of SFAM show that SFAM_mblock performed best and was the most robust for all classes of sequence similarity, thanks to its macroscopic approach that assembles PSpA blocks into multiple blocks, instead of relying on MSA. The results also show that SFAM_mblock produced MSpAs that yielded sequence alignments with accuracy higher than or comparable to that of state-of-the-art MSA methods. The power of SFAM_mblock derives from using splicing structure information (i.e. the exon–intron structure for genes and the exon–exon structure for CDSs) and the spliced alignment graph that makes it possible to consistently group conserved exon blocks of PSpAs in MSpA blocks without relying on sequence alignment. This shows the accuracy of the underlying MSpAs. This also illustrates that it is possible to obtain high-quality MSpAs in a reasonable amount of time by focusing on the splicing structure information. Some MSA methods that are agnostic to splicing structure information such as Mirage, PRANK and MACSE performed comparably to SFAM_mblock for MSA. In contrast, their performance either decreased faster when the sequence similarity increased or they were much more computationally intensive. Even if some MSA methods performed as well as SFAM_mblock and might be preferred if the splicing structure information is not available or splicing structure conservation is not important, the accurate MSAs provided by SFAM_mblock constitute robust foundations for its other applications: CDS orthology groups inference and gene-model prediction.

### 3.2 Performance for CDS orthology group inference

#### 3.2.1 Comparison based on RI

**Fig. 5.**
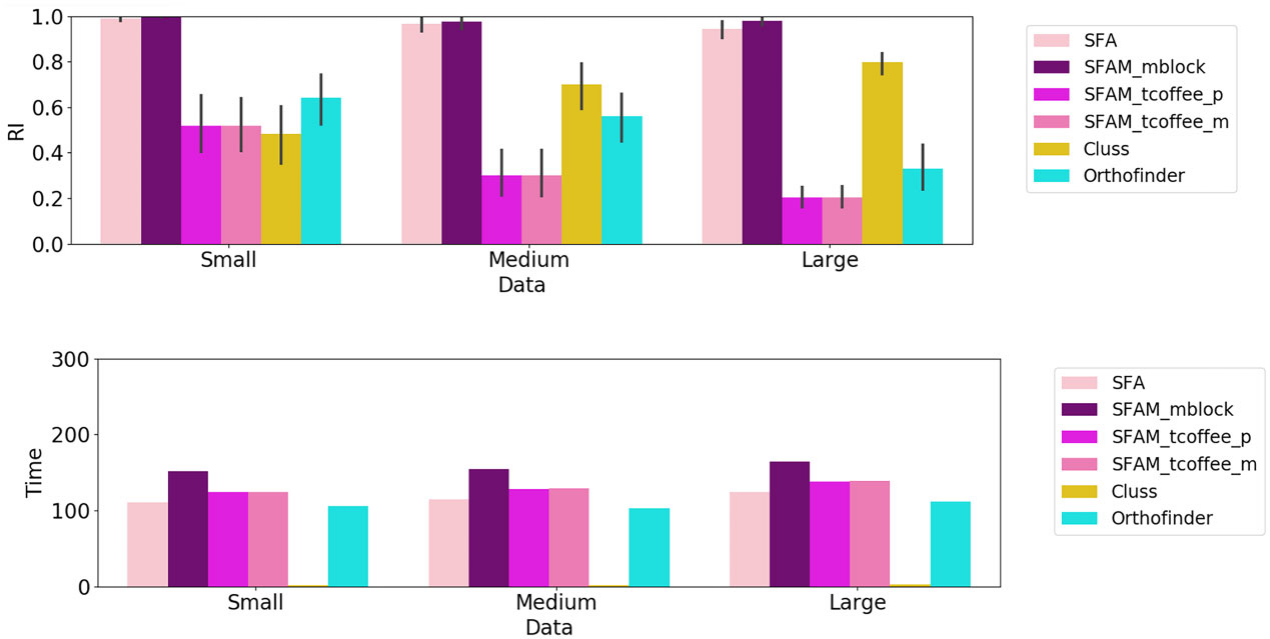
RI with standard deviations (top), and computing times (bottom) for the CDS clusters obtained using SFA, SFAM (SFAM_mblock, SFAM_tcoffe_p, SFAM_tcoffe_m), Cluss and OrthoFinder for each simulated dataset

Figure 5 shows the comparison of the RI measure of SFA, SFAM (SFAM_mblock, SFAM_tcoffee_p, SFAM_tcoffee_m), Cluss and OrthoFinder. The RI of SFA and SFAM_mblock was the highest (close to 1) for all classes of sequence similarity compared to the other methods. The RI of SFAM_tcoffee_p, SFAM_tcoffee_m and OrthoFinder decreased faster when sequence similarity decreased, while the performance of Cluss increased. It is not surprising that the performance of Cluss increased when the sequence similarity decreased, because Cluss is an alignment-free clustering method designed especially for hard-to-align sequences such as highly dissimilar sequences. The low performance of Cluss and OrthoFinder compared to SFA and SFAM_mblock for CDS orthology group inference can be explained by the fact that Cluss and OrthoFinder are not designed for at distinguishing isoforms of the same gene, since they solely rely on sequence similarity. Thus, most of the time, they put isoforms of the same gene in the same orthology groups. In contrast, SFA and SFAM_mblock are able to discriminate between isoforms of the same gene thanks to accounting for the splicing structure comparison between CDS. This illustrates the importance of taking account of splicing structural information to compare and cluster spliced sequences.

#### 3.2.2 Comparison of execution times

As for the execution times, SFA, SFAM_mblock, SFAM_tcoffee_p, SFAM_tcoffee_m and OrthoFinder had comparable running times for all classes of sequence similarity (*Small*, *Medium* and *Large*). Cluss had the lowest running time, because, unlike the other methods, it does not rely on any computationally intensive sequence or structure alignment step. Rather it relies on an alignment-free sequence similarity measure.

### 3.3 Performance in predicting gene models

Table 3 shows the precision, recall and *F*-score of the prediction of new CDS for each of the 20 real gene families in release 97 of the Ensembl database. SFAM_mblock predicted new CDSs for all 20 gene families (i.e. |P|>0). Based on the stringent method that defines two CDSs of the true and predicted CDS sets as matching if they are 100% identical, SFAM_mblock succeeded in predicting true new CDSs (present in release 98) in 10 out of the 20 gene families (i.e. $|T\cap P{|}_{100}>0$) with a precision rate up to 50% (for family *ENSGT*00390000004965), a recall rate up to 100% (for *ENSGT*00390000004965 and *ENSGT*00390000005532) and an *F*-score rate up to 67% (for *ENSGT*00390000004965). With the second less stringent method which defines two CDSs as matching if they have a PID higher than 90% and are reciprocal best hits, SFAM_mblock succeeds to predict true new CDS in 15 out of the 20 gene families (i.e. $|T\cap P{|}_{90}>0$) with a precision rate up to 50% for 3 families, a recall rate up to 100% for 2 families and an *F*-score rate up to 67% for 1 family.

**Table 3.** Precision, recall and *F*-score of the prediction of new CDS by SFAM_mblock on the 20 real gene families

Gene family (tree) ID	||P||	||T||	||T∩P|| (100)–(90)	Precision–recall– *F*-score (100)	Precision–recall– *F*-score (90)
ENSGT…3205	7	3	0–1	0–0–0	0.14–0.33–0.2
ENSGT…8371	7	1	0–0	0–0–0	0–0–0
ENSGT…0715	2	2	0–0	0–0–0	0–0–0
ENSGT…7909	10	4	0–2	0–0–0	0.2–0.5–0.29
ENSGT…3187	4	3	0–2	0–0–0	0.5–0.67–0.57
ENSGT…2978	24	13	4–5	0.17–0.31–0.22	0.21–0.38–0.27
ENSGT…2681	15	10	2–2	0.13–0.2–0.16	0.13–0.2–0.16
ENSGT…2875	10	4	0–0	0–0–0	0–0–0
ENSGT…2728	9	8	1–3	0.11–0.12–0.12	0.33–0.37–0.35
ENSGT…2931	13	10	1–2	0.08–0.1–0.09	0.15–0.2–0.17
ENSGT…2705	36	4	1–1	0.03–0.25–0.05	0.03–0.25–0.05
ENSGT…3023	9	4	0–1	0–0–0	0.11–0.25–0.15
ENSGT…3241	4	3	0–0	0–0–0	0–0–0
ENSGT…2956	19	13	0–2	0–0–0	0.11–0.15–0.12
ENSGT…2783	11	4	0–0	0–0–0	0–0–0
ENSGT…3192	22	4	2–2	0.09–0.5–0.15	0.09–0.5–0.15
ENSGT…2727	11	11	1–2	0.09–0.09–0.09	0.18–0.18–0.18
ENSGT…4965	2	1	1–1	0.5–1–0.67	0.5–1–0.67
ENSGT…3967	4	3	1–2	0.25–0.33–0.29	0.5–0.67–0.57
ENSGT…5532	4	1	1–1	0.25–1–0.4	0.25–1–0.4
Total	223	106	15–29	0.07–0.14–0.09	0.13–0.27–0.18

*Notes:* The results for the stringent method requiring 100% PID between matching CDSs, and the less stringent method requiring at least 90% identity are provided. P means predicted CDS by SFAM_mblock and T means true CDS from release 97.

Overall, with the stringent method, SFAM_mblock effectively predicted 15 out of the 106 new dog CDSs in release 98 of Ensembl but not in release 97 (i.e. recall = 14%). With the less stringent method, it predicted 29 out of the 106 new dog CDS (i.e. recall = 27%). This can be attributed to the comparison with homologous genes from only five other species. It is important to indicate that the new CDSs were findable by SFAM_mblock only if they had conserved counterparts (splicing orthologs) in homologous genes in release 97 of Ensembl. Thus, there was a proportion of new CDSs that lack splicing orthologs in homologous genes that were not targeted by SFAM_mblock, and which should be predicted using complementary methods. Yet, SFAM_mblock’s ability to predict up to 27% of the new CDSs is remarkable. It demonstrates the usefulness of SFAM_mblock predicting gene models by homology in order to improve existing genome annotations. Moreover, the number of newly annotated exons and alternative splicing transcripts is constantly increasing in databases. This indicates that there is a proportion of CDSs yet to be found. These CDSs might not be currently annotated because they contain exons that have not yet been annotated. Thus, the set of true CDSs might be larger in reality, and so would the intersection of the true and predicted CDS sets. Therefore, the predictions underestimated the precision and recall rates.

## 4 Conclusion

The ability to identify conserved gene transcripts in gene families is a fundamental step for studying the evolution of alternative transcripts. This will help identify which alternative transcripts are conserved across genes and species, and thus are the probable main functional isoforms of a gene. It will also make it possible to study the impact that new alternative transcripts have upon the function of a gene. It will offer a broader picture of the timing of gain and loss of alternative transcripts and help refine hypotheses concerning these evolutionary events.

Moreover, several ongoing projects aim to fully sequence thousands of eukaryote genomes (Lewin *et al.*, 2018). In this context, the availability of proper automated gene-model inference methods is critical gaining the most insight from these data. It is especially important because, in most eukaryote species, the coding portion of genes is divided by long introns, making identification of the actual CDSs and actual exon boundaries difficult. This work illustrates that MSpA can help identify CDSs that are conserved across multiple genes and species. A better understanding of the evolutionary history of alternative transcripts will also help in this regard, as more weight will then be given to conserved CDSs identified in the genome annotation.

This paper introduced the MSpA problems and describes the SFAM method that computes MSpAs and provides for effectively identifying conserved gene transcripts by performing CDS orthology group inference. Compared with popular MSA methods and sequence clustering methods, SFAM_mblock achieved comparable or higher performance than other methods with more robustness to changes in the sequence similarities. SFAM_mblock was also effective at predicting gene models by homology based on the computation of MSpAs. Applying SFAM_mblock to 20 real gene families from the Ensembl database revealed that up to 27% of the true new dog CDSs in release 98 could be predicted based on data from release 97.

The SFAM method takes into account both evolutionary rate and changes in the splicing structure, but does not include other events commonly discussed in gene orthology inference and evolutionary biology: duplication and losses of genes. These events are commonly the target of orthology inference methods such as OrthoFinder. Future work will define spliced homology groups in this context as proposed in Kuitche *et al.* (2017). This might be handled by generalizing the gene-tree-species-tree reconciliation framework to transcript-tree-gene-tree reconciliations (Kuitche *et al.*, 2017, 2021) to account for events that modify sets of alternative transcripts.

## Supplementary Material

vbab044_Supplementary_Data
