# Supplementary material for: From pairwise to multiple spliced alignment
Source: Bioinform Adv. 2022 Jan 5;2(1):vbab044. doi: 10.1093/bioadv/vbab044 (PMC9710695; doi:10.1093/bioadv/vbab044)
Supplement: vbab044_Supplementary_Data [file vbab044_supplementary_data.pdf]

# Supplementary Materials for: From pairwise to multiple spliced alignment

Safa Jammali, Abigaïl Djossou, Wend-Yam D. D. Ouédraogo, Yannis Nevers, Ibrahim Chegrane, and Aïda Ouangraoua

## 1 Detailed description of the SplicedFamAlignMulti Algorithms

See Supplementary Figure S1 for an overview of the methods.

### 1.1 T-Coffee-based multiple spliced alignment:

The SFAM_tcoffee algorithm is composed of three steps:

1. **Generate the primary library of residue pairs:**

   - **Library generation for the SFAM_tcoffee_p (p for pairwise) method**: The method makes directly use of the blocks of the pairwise spliced alignment given as input to SFAM_tcoffee. For any CDS $c \in \mathcal{C}$ of a gene $g \in \mathcal{G}$, and any gene $h \in \mathcal{G}$ such that $g \neq h$, we consider the pairwise spliced alignment $X$ of $c$ and $h$. For any conserved block $X[i] = \{h : (s_i^h, e_i^h), c : (s_i^c, e_i^c)\}$ of $X$, the pairwise global alignment of segments $(s_i^h, e_i^h)$ and $(s_i^c, e_i^c)$ is computed using a global alignment with affine gap costs (using the pairwise sequence alignment function *globalms* of the Python module *Bio.pairwise2,align* with parameters: 2 (match score), 0 (mismatch score), -10 (gap opening penalty), -1 (gap extension penalty)). For each pair of aligned residues $(g[c][k], h[l])$, the pair of residues is transformed into a pair of aligned residues of $g$ and $h$, i.e $(gpos_{c \to g}(k), h[l])$ which is added to the library with a weight corresponding to the percent sequence identity (PID) of the alignment. For instance, for the conserved block $W[3]$ of the pairwise spliced alignment $W$ between CDS $c_3$ and gene $g$ depicted in Figure 1(C), the following six pairs of aligned residues of $g$ and $h$ are added with a weight $\frac{2}{8}$: $(41, 46), (42, 47), (43, 48), (44, 51), (45, 52), (46, 53)$.
   - **Library generation for the SFAM_tcoffee_m (m for multiple) method**: The method makes use of the alignment graph $graph(\mathcal{X})$. For each connected component $cc$ of $graph(\mathcal{X})$, a multiple sequence alignment is computed for the set of gene segments contained in $cc$ (using the multiple sequence aligner MAFFT through the function *MafftCommandline* of the Python module *Bio.Align.Applications* with

parameters: localpair=True (pairwise alignments computed with the Smith-Waterman algorithm) and lop=-6.02 (gap opening penalty for pairwise alignments)). For any block edge contained in $cc$ between a gene segment $(s_i^h, e_i^h)$ and a CDS segment $(s_i^c, e_i^c)$, we consider the pairwise alignment of $(s_i^h, e_i^h)$ and $(gpos_{c\rightarrow g}(s_i^c), gpos_{c\rightarrow g}(e_i^c))$ induced by the multiple sequence alignment. Each pair of aligned residues in the pairwise alignment is added to the library with a weight that is the PID of the pairwise alignment. For instance, for the block edge $(g : (9, 12), h[c3] : (1, 4))$ in Figure 2, the pairs of aligned residues of $g$ and $h$, $(9, 8), (10, 9), (11, 10), (12, 11)$, are added with a weight 3/4.

2. **Compute a multiple sequence alignment $M$ of $\mathcal{G}$**: Using the T-Coffee algorithm with default parameters and the library of residue pairs computed at the previous step, a multiple sequence alignment of all gene sequences of $\mathcal{G}$ is computed;

3. **Compute a multiple spliced alignment of $\mathcal{C} \cup \mathcal{G}$ given the multiple sequence alignment $M$ of $\mathcal{G}$**: The set of all segments of all gene structures is partitioned such that for any pair of genes $(g, h) \in \mathcal{G}^2$, and any pair of segments $(\mathcal{S}(g)[i], \mathcal{S}(h)[j])$ from the gene structures of $g$ and $h$, the segments $\mathcal{S}(g)[i]$ and $\mathcal{S}(h)[j]$ are included in the same group if:

   - More than half of the residues of segment $\mathcal{S}(g)[i]$ are aligned with residues of segment $\mathcal{S}(h)[j]$; or
   - There exist $i_1$ and $i_2$ such that $1 \leq i_1 < i < i_2 \leq \mid \mathcal{S}(g) \mid$, and $\mathcal{S}(g)[i_1]$ and $\mathcal{S}(g)[i_2]$ are included in the same multi-block as $\mathcal{S}(h)[j]$;

   If a group contains multiple segments of a gene structure $\mathcal{S}(g)$, these segments are merged into a single segment of $g$ whose start location (resp. end location) is the minimum start (resp. maximum end) location of all these segments. Each resulting group is then defined as a multi-block of the multiple spliced alignment. For instance, given the following alignment of genes $g$ and $h$ from Figure 1(A),

```
g : ***ATGGAATGC****----****AAGCAG----GTCTGG****ACGTGG****GG---TGATTGA***
h : ***---------****ATGA****ATGCCG****GTAACG**************GACTTTGAATAA***
```

   the following five groups of segments are computed {{g:(4,12)}, {h:(8,11)}, {g:(25,36), h:(16,21), h:(26,31)}, {g:(41,46)}, {g:(51,59), h:(46,57)}}, and the resulting multiple spliced alignment is depicted in Figure 1(B).

## 1.2 Graph-based multiple spliced alignment:

The SFAM_mblock is composed of four steps:

1. **Compute the alignment graph** $graph(\mathcal{X})$;

2. **Weight the edges of** $graph(\mathcal{X})$ : To each edge $e$ of $graph(\mathcal{X})$, we assign two scores denoted by $PID(e)$ and $connect(e)$ that are confidence scores for the segment alignment represented by $e$. For any block edge $e$ between a gene segment $(s_i^h, e_i^h)$ and a CDS segment $(s_i^c, e_i^c)$ :

- $PID(e)$ is the PID of the pairwise alignment of segments $(s_i^h, e_i^h)$ and $(s_i^c, e_i^c)$. Thus, we have $0 \leq PID(e) \leq 1.0$.
- $connect(e)$ is the number of block edges that belong to a shortest path between vertices $(s_i^h, e_i^h)$ and $(s_i^c, e_i^c)$ if the edge $e$ is removed. By convention, if there exists no path between $(s_i^h, e_i^h)$ and $(s_i^c, e_i^c)$ after the removal of $e$, then $connect(e) = m$ such that $m$ is the total number of block edges in $graph(\mathcal{X})$ (see Figure 2 for an illustration). Thus, for any block edge $e$, we have $1 \leq connect(e) \leq m$. $connect(e)$ reflects how far the vertices $(s_i^h, e_i^h)$ and $(s_i^c, e_i^c)$ remain connected after the removal of $e$.

For any CDS edge $e$ between a CDS segment $(s_i^c, e_i^c)$ and the corresponding gene segment $(gpos_{c \to g}(s_i^c), gpos_{c \to g}(e_i^c))$, $PID(e) = 1.0$ and $connect(e) = 0$. For any edge $e$ of $graph(\mathcal{X})$, a high value for $PID(e)$ or a low value of $connect(e)$ provides a strong support for the segment alignment represented by edge $e$.

3. **Split connected components of** $graph(\mathcal{X})$: For each connected component $cc$ of $graph(\mathcal{X})$, if $cc$ contains two vertices $(s_{i1}^x, e_{i1}^x)$ and $(s_{i2}^x, e_{i2}^x)$ corresponding to two non-overlapping segments of the same sequence $x$, a set of low confidence edges are removed from $cc$ in such a way to disconnect the two vertices. The procedure is as follows: as long as the two vertices are connected, iteratively find a shortest path between them, and remove an edge $e$ that first maximizes $connect(e)$ and then minimizes $PID(e)$. The rationale behind this step is that a multi-block must contain at most one segment of each sequence. Therefore, a connected component containing two non-overlapping segments of the same sequence cannot represent a multi-block. The result of this step is a new graph denoted $graph'(\mathcal{X})$. For instance, in Figure 2, the edge $(g : (41, 46), h[c3] : (17, 24))$ will be removed in order to disconnect vertices $g : (41, 46)$ and $g : (51, 55)$.

4. **Consider connected components of** $graph'(\mathcal{X})$ **as candidate multi-blocks, and build the multiple spliced alignment in a progressive manner**: For each connected component $cc$ of $graph'(\mathcal{X})$, a candidate multi-block composed of the segments (vertices) in $cc$ is built. The resulting set $\mathcal{M}$ of candidate multi-blocks is ordered by decreasing multi-block size. The multiple spliced alignment $A$ is initialized to an empty chain. At each iteration until $\mathcal{M}$ is empty, the first multi-block $a \in \mathcal{M}$ is removed. If $a$ is consistent with $A$ then $a$ is added to $A$, otherwise a minimum number of gene segments with their corresponding CDS segments are removed from $a$ to make the latter consistent with $A$. Then, the resulting multi-block, that has a lower size than $a$, is added to $\mathcal{M}$ while preserving the order of multi-blocks by decreasing size in $\mathcal{M}$.

   For instance, in Figure 2, $graph'(\mathcal{X})$ (obtained after removing the edge $(g : (41, 46), h[c3] : (17, 24))$) contains ten connected components numbered from 1 to 10 by decreasing size. The candidate multi-blocks (1), (2), and (3) are first found consistent with $A$ and added to the spliced alignment to yield two multi-blocks in $A$, (1)+(3) = {g:(25,36), g[c1]:(5,16), g[c2]:(10,15), h:(16,31), h[c3]:(5,16), h[c4]:(1,6)}, and (2) = {g:(9,12), g[c1]:(1,4), h:(8,11), h[c3]:(1,4)}. Next, the candidate multi-block (4) is inconsistent with $A$, so the segments of gene $h$ and its CDS are removed from the multi-block to yield the new multi-block (4') = {g:(31,36),

g[c1]:(11,16)} that is added to $\mathcal{M}$. Next, the candidate multi-blocks (5) and (6) are found consistent with $A$ and added to the spliced alignment to yield a third multi-block in $A$, (5)+(6) = {g:(51,59), g[c1]:(17,21), g[c2]:(22,30), h:(46,57), h[c3]:(17,24), h[c4]:(7,18)}. Next, the candidate multi-block (7) is inconsistent with $A$, so the segments of gene $h$ and its CDS are removed to yield the multi-block (7') = {g:(25,35)} that is added to $\mathcal{M}$. Next, the candidate multi-blocks (8), (9) and (10) are found consistent with $A$ and added to yield the new multi-block (8)={g:(41,46), g[c2]:(16,21), h:(36,41)}, and an extension of multi-block (2) into (2)+(10) = {g:(4,12), g[c1]:(1,4), g[c2]:(1,9), h:(8,11), h[c3]:(1,4)}. The multi-block (5)+(6) becomes (5)+(6)+(9) and remains unchanged. Finally, the modified multi-blocks (4′) and (7′) are added in multi-block (1)+(3) without any modification of the segments. The final spliced alignment is depicted in Figure 3(A).

# 2 Supplementary figure

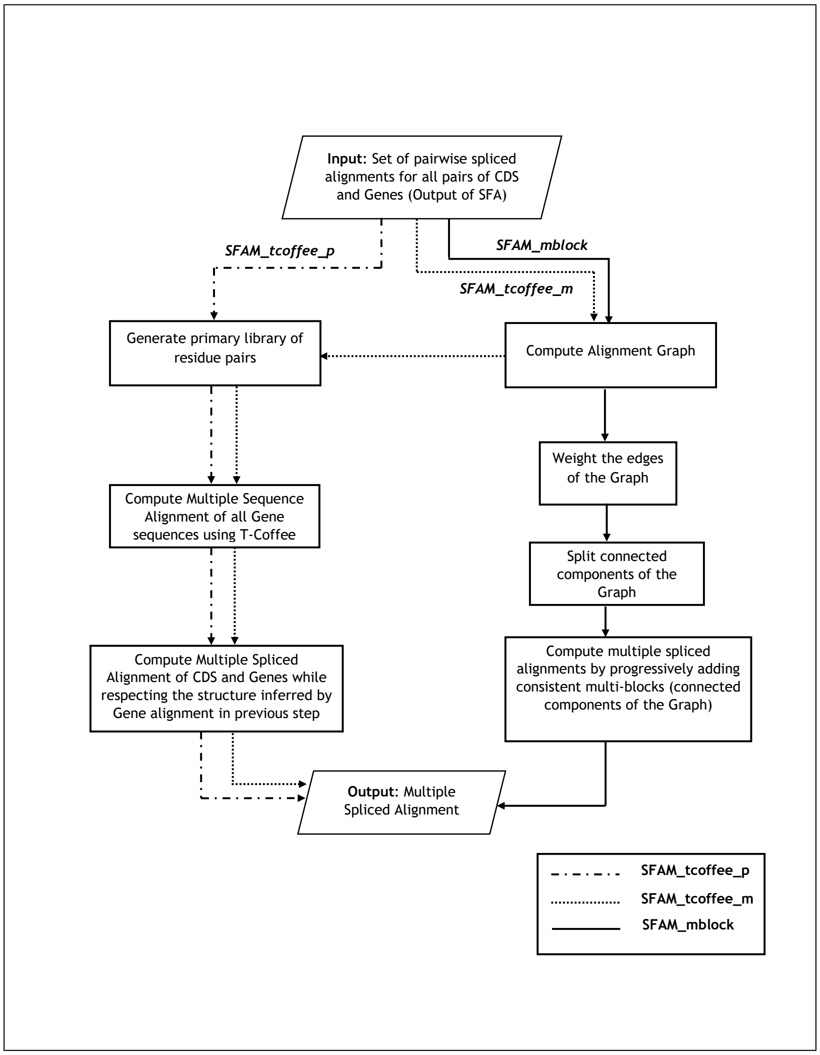


Figure 1: Overview of SpliceFamAlignMulti methods
